# Supplementary material for: Content-rich biological network constructed by mining PubMed abstracts
Source: BMC Bioinformatics. 2004 Oct 8;5:147. doi: 10.1186/1471-2105-5-147 (PMC528731; doi:10.1186/1471-2105-5-147)
Supplement: Additional File 2 — The original results of the above study (non-essential files are deleted to keep the file size under the limit set by BMC bioinformatics). [file 1471-2105-5-147-S2.bz2 › chilibotAdditionalFile2/dip40/127ID8609467E471/html/P_N.html]

 


 **P** and **N** 
  
Found 149281 abstracts in PubMed, retrieved 40.  
 

 What does Google say? 
 PDF only 
| .edu only 

---

**Interactive relationship** (e.g. stimulation, inhibition, etc)

**Neutral relationship**- After 5 days of oral treatment with 10 mg kg of the drug, the average mass of the tumors in treated mice 198 42 mg,  **n**  = 5 was significantly  **p**  <.  Ref: 12866022 Int J Cancer, 2003
- These reactions led to the new spiro closo carboranylphosphazenes gem  **N**  3  **P**  3 Cl 6 2n OCH 2 2 C 2 B 10 H 10  **n**   **n** =1 2 and the first fully carborane substituted phosphazene gem  **N**  3  **P**  3 OCH 2 2 C 2 B 10 H 10 3 3.  Ref: 12866075 ChemistryChemistry, 2003
- Breast cancer tissues  **n**  = 120 exhibited a lower level of PPARgamma mRNA compared to normal tissues  **n**  = 25,  **p**  = 0.05.  Ref: 12866036 Int J Cancer, 2003
- However, the reaction of gem  **N**  3  **P**  3 O 2 C 12 H 8 2 OCH 2 2 C 2 B 10 H 10 5 with NEt 3 in ethanol instead of acetone proceeded in a different manner to give the new compound NHEt 3 2  **N**  3  **P**  3 O 2 C 12 H 8 2 O OCH 2 C 2 B 9 H 10 CH 2 OCH 2 CH 3 7.  Ref: 12866075 ChemistryChemistry, 2003

**Non-interactive relationship** (e.g. studied together, co-existance, homology, etc.)

- The reactions of  **N**  3  **P**  3 Cl 6 with one, two, or three equivalents of the difunctional closo carborane C 2 B 10 H 10 CH 2 OH 2 and K 2 CO 3 in acetone have been investigated.  Ref: 12866075 ChemistryChemistry, 2003
- Treatment of tert cyclobutanols with arylating reagents in toluene in the presence of Pd OAc 2, a chiral ferrocene containing  **N** ,  **P**  bidentate ligand, and Cs 2 CO 3 affords optically active gamma arylated ketones in excellent yields with high enantioselectivity up to 95% ee.  Ref: 12862483 J Am Chem Soc, 2003
- A novel enantioselective C C bond cleavage has been achieved using palladium catalysts and chiral  **N** ,  **P**  bidentate ligands in the asymmetric arylation, vinylation, and allenylation of tert cyclobutanols.  Ref: 12862483 J Am Chem Soc, 2003
- SigmaPCBlw in WB was the parameter that best described the variation in PL retinol concentration r 2 =0.455,  **n** =20,  **P** =0.0007.  Ref: 12865050 Environ Res, 2003
- However, for 5 and 7 only the meso compounds seem to be formed, with the same R, S configuration as in the precursor  **N**  3  **P**  3 Cl 2 O 2 C 12 H 8 2.  Ref: 12866075 ChemistryChemistry, 2003
- The reaction of the well known spiro derivatives  **N**  3  **P**  3 Cl 2 O 2 C 12 H 8 2 and  **N**  3  **P**  3 Cl 4 O 2 C 12 H 8 with the same carborane diol and K 2 CO 3 in acetone gave the new compounds gem  **N**  3  **P**  3 O 2 C 12 H 8 3  **n**  OCH 2 2 C 2 B 10 H 10  **n**   **n** =1 5 or 2 6, respectively, without signs of intra or intermolecularly bridged species.  Ref: 12866075 ChemistryChemistry, 2003
- A bridged product, non gem  **N**  3  **P**  3 Cl 4 OCH 2 2 C 2 B 10 H 10 4, was also detected.  Ref: 12866075 ChemistryChemistry, 2003
- controls  **n** =458,  **P** < ; 0.001.  Ref: 12865131 Neurosci Lett, 2003
- Together with the relationships between rPPFD and  **N**  on a mass basis r 2 = 0.80 and SLA and V cmax r 2 = 0.60, these findings suggest that most light acclimation in  **P** . densiflora occurs through changes in needle morphology e.g., SLA during development.  Ref: 12865251 Tree Physiol, 2003
- **n**  = 20 for the ODI and 0.95  **P**  <.  Ref: 12865852 Spine, 2003
- Ethnic differences in STH genotype frequencies for cases and controls were observed and these were statistically significant cases  **n** =344,  **P** < ; 0.03.  Ref: 12865131 Neurosci Lett, 2003
- Compounds in which quadruply bonded Mo 2 4 units, Mo 2 DAniF 3 DAniF =  **N** ,  **N**  di  **p**  anisylformamidinate, are linked by cyclic diamidate anions have been synthesized and characterized by X ray crystallography and spectroscopic methods.  Ref: 12862486 J Am Chem SocJ Am Chem Soc, 2003
- For compounds with two dioxybiphenyl units, gem  **N**  3  **P**  3 O 2 C 12 H 8 2 OCH 2 2 C 2 B 10 H 10 5, NHEt 3  **N**  3  **P**  3 O 2 C 12 H 8 2 OCH 2 2 C 2 B 9 H 10 8, and NHEt 3 2  **N**  3  **P**  3 O 2 C 12 H 8 2 O OCH 2 C 2 B 9 H 10 CH 2 OCH 2 CH 3 7, a mixture of different stereoisomers may be expected.  Ref: 12866075 ChemistryChemistry, 2003
